# Thermostability Engineering of a Class II Pyruvate Aldolase from *Escherichia coli* by *in Vivo* Folding Interference

**DOI:** 10.1021/acssuschemeng.1c00699

**Published:** 2021-04-07

**Authors:** Sandra Bosch, Esther Sanchez-Freire, María Luisa del Pozo, Morana C̆esnik, Jaime Quesada, Diana M. Mate, Karel Hernández, Yuyin Qi, Pere Clapés, Đurđa Vasić-Rački, Zvjezdana Findrik Blažević, José Berenguer, Aurelio Hidalgo

**Affiliations:** † Department of Molecular Biology, Center of Molecular Biology “Severo Ochoa” (UAM-CSIC), Autonomous University of Madrid, Nicolás Cabrera 1, 28049 Madrid, Spain; ‡ University of Zagreb, Faculty of Chemical Engineering and Technology, Savska c. 16, HR-10000 Zagreb, Croatia; ∥ Institute of Advanced Chemistry of Catalonia, Biotransformation and Bioactive Molecules Group, Spanish National Research Council (IQAC−CSIC), Jordi Girona 18-26, 08034 Barcelona, Spain; § Prozomix Ltd., Station Court, Haltwhistle, NE49 9HN Northumberland, United Kingdom

**Keywords:** aldolase, directed evolution, hygromycin B phosphotransferase, *in vivo* selection, thermostability, *Thermus thermophilus*

## Abstract

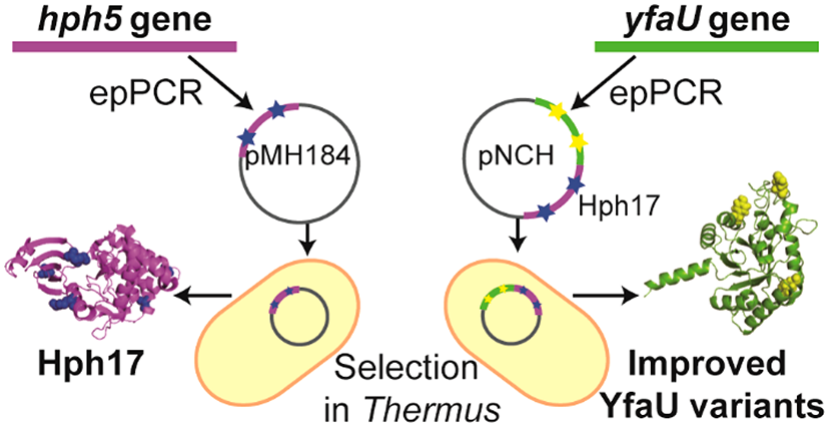

The use of enzymes in industrial processes is often limited by the unavailability of biocatalysts with prolonged stability. Thermostable enzymes allow increased process temperature and thus higher substrate and product solubility, reuse of expensive biocatalysts, resistance against organic solvents, and better “evolvability” of enzymes. In this work, we have used an activity-independent method for the selection of thermostable variants of any protein in *Thermus thermophilus* through folding interference at high temperature of a thermostable antibiotic reporter protein at the C-terminus of a fusion protein. To generate a monomeric folding reporter, we have increased the thermostability of the moderately thermostable Hph5 variant of the hygromycin B phosphotransferase from *Escherichia coli* to meet the method requirements. The final Hph17 variant showed 1.5 °C higher melting temperature (*T*_m_) and 3-fold longer half-life at 65 °C compared to parental Hph5, with no changes in the steady-state kinetic parameters. Additionally, we demonstrate the validity of the reporter by stabilizing the 2-keto-3-deoxy-l-rhamnonate aldolase from *E. coli* (YfaU). The most thermostable multiple-mutated variants thus obtained, YfaU99 and YfaU103, showed increases of 2 and 2.9 °C in *T*_m_ compared to the wild-type enzyme but severely lower retro-aldol activities (150- and 120-fold, respectively). After segregation of the mutations, the most thermostable single variant, Q107R, showed a *T*_m_ 8.9 °C higher, a 16-fold improvement in half-life at 60 °C and higher operational stability than the wild-type, without substantial modification of the kinetic parameters.

## Abstract 2

This work demonstrates a method for selecting thermostable variants of any protein of interest to be used in biocatalysis, increasing the sustainability of industrial chemical transformations.

## Introduction

Reaction conditions of enzymes in industrial biocatalysis are usually far from those in nature: non-natural substrates are used in high concentrations while higher temperatures and organic cosolvents are needed to promote substrate and product solubility. In this context, enzyme engineering constitutes an efficient methodology to tailor enzyme activity, substrate selectivity, or stability under operational conditions to each industrial process.^1^

The rational prediction of thermostability is a complex task because methods are based on different structure–function hypotheses, leading to different solutions, which in many cases do not result in direct increases in stability.^2^ Therefore, directed evolution is preferred, since it allows exploration of a large sequence space (in the range of 10^6^ to 10^9^ individuals),^3^ albeit at the cost of increasing the screening effort to cover a meaningful fraction of this man-made diversity.

Screening for thermostable enzyme variants in large libraries can be carried out in a thermophile, provided its growth is coupled to the stability of the target protein.^4^ In 2007, we reported a procedure for the *in vivo* selection of thermostable variants of any protein independently of its activity using *Thermus thermophilus* as a host.^5^ The method was based on the folding interference phenomenon that occurs in a protein fusion between a thermosensitive target protein in the N-terminus and a thermostable kanamycin nucleotidyl transferase^4^ (Kat) in the C-terminus (Figure S1). This method has proven useful for the isolation of thermostable variants of human interferons and enzymes for biocatalysis, such as lipase A from *Bacillus subtilis*, formate dehydrogenase from *Pseudomonas* sp. 101,^5^ and more recently, the esterase I from *Pseudomonas fluorescens*.^6^

In the course of generating thermostable variants of the latter enzyme, we encountered a large number of false positives that we attributed to having used a dimeric folding interference reporter, such as Kat. Therefore, we evolved the monomeric, moderately thermostable hygromycin B phosphotransferase variant (Hph5) from *Escherichia coli* reported by Nakamura et al.^7^ Hph5 accumulated five amino acid substitutions that allowed *T. thermophilus* to grow at temperatures up to 67 °C. However, lower transformation efficiency of this marker in *Thermus* had been reported at that temperature,^7^ compromising the throughput of our selection method as well as limiting the selection pressure, i.e. temperature, that can be applied.

Consequently, in this work we engineered a bespoke, highly thermostable, monomeric folding reporter (Hph17) and used it to stabilize the *E. coli* 2-keto-3-deoxy-l-rhamnonate aldolase (YfaU). YfaU is a class II pyruvate aldolase that accepts a wide range of electrophiles, and even though the natural nucleophilic substrate is pyruvate, it can also use homologous ketoacids. The aldol addition of pyruvate or homologues to a wide variety of *N*-carboxybenzyl-amino aldehydes are especially relevant since the resulting aldol adducts are intermediates of new proline, pyrrolizidine-3-carboxylic acid, pipecolic acid, and β-hydroxy-γ-amino acid derivatives.^8,9^ Moreover, YfaU plays an important role in the biocatalytic cascade for the synthesis of the noncanonical amino acid (*S*)-2-amino-4-hydroxybutanoic acid (l-homoserine). YfaU can synthesize (*S*)- or (*R*)-2-amino-4-hydroxybutanoic acid with *ee* values of >99% using pyruvate and formaldehyde as substrates, and a transaminase provides pyruvate from alanine, thus l-homoserine is produced using formaldehyde and alanine as sole and inexpensive starting materials^10^

## Results and Discussion

### Library Generation and Selection of Hph Variants

In order to improve the stability of Hph5 for its use as a folding interference reporter, the *hph5* gene was randomized by error-prone PCR (epPCR) in the presence of 0.2 mM Mn^2+^ to introduce 3–6 nucleotide replacements per gene, which represent between 2 and 5 amino acid changes, in good agreement with most directed evolution studies.^11^ The epPCR Hph5 library was generated in *E. coli* and then transformed in *T. thermophilus* for selection. The generated *E. coli* library of 4.5 × 10^4^ individuals was selected at 70 °C and 100 μg/mL of hygromycin B (HygB), at which transformants expressing parental Hph5 could not grow (Figure S2). Under permissive conditions (60 °C and 100 μg/mL of HygB), 9961 CFU/ng plasmid were obtained, while under selection pressure (70 °C and 100 μg/mL of HygB) only 32 CFU/ng of plasmid were selected, which represents a selection factor of 0.32%. Because of the high number of transformants obtained under those conditions, the temperature had to be subsequently increased to 71 °C, leading to 2 CFU/ng plasmid and a selection factor of 0.02%.

Twenty randomly selected clones were verified for HygB resistance using a serial dilution assay at 71 °C (Figure S3, A). A particular variant (Hph17) harboring five changes (R61H, S86G, Q96P, A185V, and V322E) was found four times in the pool and enabled growth of *Thermus* even at 74 °C (Figure S3B). It seems unlikely that all of these four individuals originated independently during epPCR, but their recurrence is likely a natural consequence of library construction in *E. coli* prior to selection in *Thermus*. Most importantly, unlike the *in vivo* mutagenesis used by Nakamura to generate Hph5,^7^*in vitro* mutagenesis by epPCR likely enabled the creation of a larger and more diverse sequence space, from which a fitter variant can be selected. In fact, it took a combination of natural evolution, DNA shuffling, and random amino acid duplications to confer a similar degree of thermostability to a hygromycin phosphotransferase from *Streptomyces hygroscopicus* (Hyg10).^12^

### Kinetic, Thermodynamic, and Structural Characterization of Hph17

**Figure 1:**
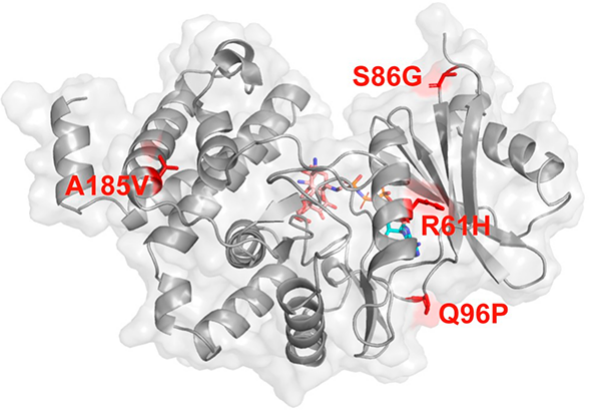
Location of stabilizing mutations in Hph17 variant. Amino acid substitutions are shown as red sticks. Substrates hygromycin B (HygB) and phosphoaminophosphonic acid-adenylate ester (ANP) are depicted as sticks in CPK colors with carbon atoms in salmon and cyan, respectively.

The mutations of the moderately thermostable Hph5 were mostly situated in the hydrophobic core.^13^ In contrast, three out of the four thermostabilizing positions mutated in the Hph17 variant (R61H, S86G, Q96P, and A185V) are found on the protein surface, except A185V, which is located in a hydrophobic core (Figure 1), reducing the distance between the adjacent β-strands and contributing toward compactness.^14^ On the other hand, residue 96 is placed in a loop and substitution of Gln to Pro in a loop diminishes the RMSD of that region, contributing to the overall stabilization of the enzyme. Finally, A185V strengthens the hydrophobic interactions and increases the protein packing^3^ since Val is bulkier than Ala.

**Figure 2:**
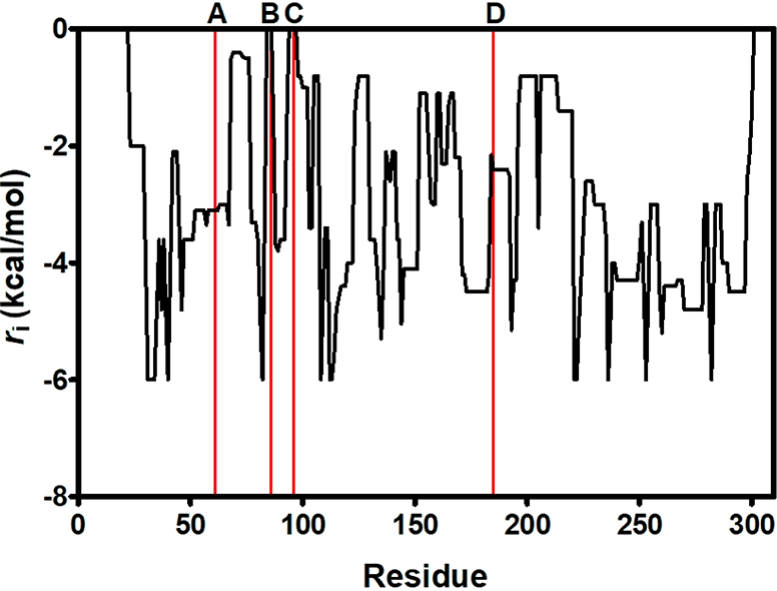
Constraint network analysis of Hph5. Lines indicate the four amino acid replacements in Hph17 (line A corresponds to position Arg61; line B, Ser86; line C, Gln96; and line D, Ala185) that can be mapped in the homology model.

As shown in Table 1, both the catalytic constant, *k*_cat_, and the efficiency for ATP, *K*_M,ATP_, remained unaltered in Hph17 compared with the parental enzyme. However, *K*_M,HygB_, was 2.4-fold higher for Hph17 respect to that of Hph5, with a consequent reduction in the catalytic efficiency. Regarding thermostability, the melting temperature of Hph17 was 1.5 °C higher than that of Hph5, while its half-life at 65 °C doubled, with the main contributions toward this enhancement originating from replacements S86G and Q96P. Increases in kinetic stability usually suggest that these mutations may interfere with an initial step on the path toward the irreversible unfolded state, thus avoiding further global unfolding.^15,16^ Therefore, we used constraint network analysis (CNA), to simulate protein unfolding.^17^ As shown in Figure 2, positions Ser86 and Gln96 were some of the most flexible *loci* in the protein (highest *r*_i_ values), congruently with the postulated “hinge” function of neighboring Val98.^13^ Thus, replacing Ser86 and Gln96 would restrict local movements leading to unfolded states by irreversible denaturation, which might explain the increase in half-life of variants S86G and Q96P (Table 1).

**Table 1:** Kinetic^a^ and Stability Parameters for Hph5, Hph17, and the Segregated Variants Containing the Amino Acid Replacements of Hph17^b^

Hph variant	*K*_M,HygB_ (mM)	*K*_M,ATP_ (mM)	*k*_cat_ (min^–1^)	*k*_cat_/*K*_M,HygB_ (min^–1^ mM^–1^)	*k*_cat_/*K*_M,ATP_ (min^–1^ mM^–1^)	*T*_m_ (°C)	half-life (min)	*k*_d_ (min^–1^)
Hph5	0.29 ± 0.03	0.37 ± 0.02	2984 ± 72	10290	8065	58.2 ± 0.1	2.2 ± 0.4	0.33 ± 0.06
Hph17	0.7 ± 0.10	0.39 ± 0.03	2847 ± 153	4067	7300	59.7 ± 0.1	7 ± 3	0.12 ± 0.05
R61H	0.84 ± 0.09	0.46 ± 0.04	3578 ± 133	4260	7778	59.5 ± 0.1	2.2 ± 0.2	0.31 ± 0.03
S86G	0.50 ± 0.07	0.55 ± 0.03	3202 ± 44	6404	5822	59.6 ± 0.3	4.7 ± 0.9	0.15 ± 0.04
Q96P	0.41 ± 0.03	0.31 ± 0.03	3555 ± 76	8671	11468	59.5 ± 0.2	3.0 ± 0.1	0.23 ± 0.01
A185V	0.39 ± 0.06	0.32 ± 0.02	2672 ± 108	6851	8350	57.9 ± 0.1	1.6 ± 0.2	0.43 ± 0.06
V322E	0.57 ± 0.09	0.39 ± 0.02	3196 ± 145	5607	8195	59.1 ± 0.4	1.9 ± 0.4	0.38 ± 0.08

a Steady-state kinetic constants were determined at 60 °C.

b Half-lives and deactivation constants (*K*_d_) were determined at 65 °C. Values represent the mean ± standard deviation of three independent determinations.

When Hph5 was evolved from Hph, a marked increase in thermodynamic stability was observed, despite the lack of a clear structural explanation.^13,18^ However, neither Hph17 nor the individual variants showed an increase in melting temperature (*T*_m_) over the parental Hph5, suggesting that a further increase of protein rigidity significant enough to gain thermodynamic stability could be detrimental for the enzyme activity. This result is not incompatible with the putative higher protein stability *in vivo*, which could be enhanced by factors such as the molecular crowding and compatible solutes of the *Thermus* cytoplasm^19^ whereas *T*_m_ determinations are carried out with the protein in buffer. In contrast, different scaffolds, such as Hyg10, have been evolved to both higher *T*_m_ (12.2 °C) and specific activity (2-fold) at the optimum activity temperature.^12^ However, the sequence identity of the Hyg10 and Hph proteins is approximately 30%, and their activity is not identical, as Hyg10 phosphorylates HygB in a different hydroxyl group.

The folding free energy of the mutants (ΔΔ*G*) was estimated using FoldX. The ΔΔ*G* values obtained were 0.78, 0.50, −0.92, 0.21, and 0.58 kcal/mol for the single variants R61H, S86G, Q96P, A185V, and the multiple mutant containing R61H, S86G, Q96P, and A185V, respectively. In this case, FoldX cannot predict correctly the found mutants given the very low differences in *T*_m_ between variants, and the standard deviation of predicted values (between 1.0 and 1.7 kcal/mol).^2^

### Library Generation and Selection of Thermostable YfaU Variants

**Figure 3:**
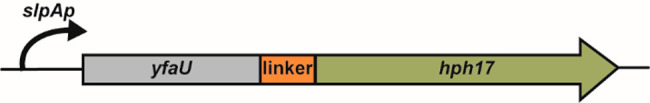
Gene fusion of *yfaU* to *hph17*, expressed under the promoter slpA (*slpAp*).

Hph17 was used as a folding interference reporter to engineer higher stability in YfaU (Figures 3 and S1). The class II pyruvate aldolase, YfaU, was mutagenized by epPCR in the presence of 0.3 mM Mn^2+^. The library sequences analyzed contained between 1 and 8 nucleotide replacements, i.e., 1–6 amino acid substitutions. The generated library of 1.5 × 10^5^ individuals was selected in *T. thermophilus* at 67 °C and 100 μg/mL of HygB, conditions under which the transformants expressing the wild-type YfaU (YfaU-wt) could not grow (Figure S4).

Due to the large number of variants selected, 54 unique clones were randomly picked to perform a dilution assay on plate at 67 °C (Figure S5). The 12 variants with the highest growth (variants 2, 8, 14, 15, 48, 50, 63, 66, 70, 99, 103, and 105) were chosen for subsequent sequencing and characterization.

### Characterization of Thermostable YfaU Variants

The 12 selected YfaU variants and YfaU-wt were cloned into pET28b, transformed in *E. coli* BL21, and expressed using autoinduction medium at 20 °C. The solubility of these variants was checked by SDS-PAGE; supernatant and pellet were run separately (Figure S6). Only four of the variants showed the presence of the protein in the supernatant fraction. The lack of solubility of these putative thermostable YfaU variants could arise from differences between the context in which they were selected and produced,^27^ i.e., a fusion protein in a thermophile host vs a standalone protein in a mesophile. Also, the low solubility of YfaU has been previously described, requiring expression in fusion with either dihydrofolate reductase (DHFR) or maltose binding protein (MBP) at the N-terminus.^10^

These four YfaU variants and YfaU-wt were purified by immobilized metal affinity chromatography (IMAC), and their *T*_m_ values were measured. Variants 2 (H49Q and G118D) and 14 (G39D and I73F) showed *T*_m_s 8.5 and 5.5 °C lower than YfaU-wt, while variants 99 (L4F, G90S, Q107R, Q141L, F215L, A252E, F254I, and I263 K) and 103 (V122F, P187T, and P261Q) increased their *T*_m_s by 2.0 and 2.9 °C, respectively, compared with YfaU-wt. However, the assessment of variants 99 and 103 using a straightforward retro-aldol reaction showed a 150- and 120-fold reduction in activity, respectively (Table 2). These results agree with previous studies of randomized libraries, in which an increase in thermal stability resulted in lower activity,^20,21^ due to a gradual loss of flexibility as the number of mutations increases.^22^ Furthermore, selection by folding interference is an activity-independent process, which may be convenient in cases where a functional selection is either complex or impossible^5^ but, in this case, led to lower activity values due to lack of selective pressure towards function.

**Table 2:** Specific Retro-Aldol Activity,^a^*T*_m_, and Half-Life^b^ of YfaU Variants

YfaU variant	specific retro-aldol activity (U/mg)	*T*_m_ (°C)	half-life (min)
wild-type	60 ± 1	60.5 ± 0.0	0.9 ± 0.1
YfaU99	0.4 ± 0.2	62.5 ± 1.0	nm
YfaU103	0.5 ± 0.3	63.4 ± 0.2	nm
Q107R	48 ± 7	69.4 ± 0.2	14 ± 1
Q141L	62 ± 6	62.7 ± 0.2	3.0 ± 0.3

a Specific activity was determined at 25 °C.

b Half-life was measured at 60 °C. Values represent the mean and the standard deviation of three independent determinations. nm: nonmeasurable.

To remediate the observed activity–stability trade-off, the amino acid replacements of these two variants were segregated and their *T*_m_s and specific activities were measured individually (Table 2). Only variants Q107R and Q141L (both derived from variant 99) increased their *T*_m_s by 8.9 and 2.2 °C, respectively, compared to YfaU-wt while increasing or maintaining the retro-aldol activities of the wild-type enzyme. In addition, the half-lives of Q107R and Q141L were 16- and 3.3-fold higher compared to YfaU-wt, respectively. Considering that only 0.01–0.5% of random mutations are beneficial,^23^ the increase in thermostability of Q107R seems to arise from a truly beneficial mutation, and the rest of the mutations in variant 99 have a deleterious or neutral effect on enzyme stability.

### Performance of YfaU Q107R and Q141L in the Aldol Addition of Pyruvate to Formaldehyde

To test the proficiency of the best YfaU variants in a biocatalytically relevant reaction, the aldol addition of pyruvate to formaldehyde was assayed, modeled and the steady-state kinetic parameters were calculated for YfaU-wt, Q107R, and Q141L (Table 3 and Figure S7).

**Table 3:** Steady-State Kinetic Parameters for the Aldol Addition of Pyruvate to Formaldehyde Catalyzed by YfaU-wt, Q107R, and Q141L^a^

YfaU variant	*k*_cat_ (min^–1^)	*K*_M,formaldehyde_ (mM)	*k*_cat_/*K*_M,formaldehyde_ (min^–1^ mM^–1^)	*K*_i,formadehyde_ (mM)	*K*_M,pyruvate_ (mM)	*k*_cat_/*K*_M,pyruvate_ (min^–1^ mM^–1^)	*K*_i,pyruvate_ (mM)
wild-type	113 ± 40	24 ± 4	4.71	95 ± 14	209 ± 83	0.54	47 ± 18
Q107R	180 ± 53	26 ± 6	6.92	75 ± 16	61 ± 20	2.95	46 ± 15
Q141L	242 ± 28	17 ± 2	14.2	113 ± 12	66 ± 10	3.67	151 ± 22

a Values represent the mean and the standard deviation of three independent determinations.

The ca. 2-fold increase in *k*_cat_ and decrease of K_M_ for both substrates for variant Q141L resulted in a 3.1- and 6.8-fold increase in catalytic efficiency (*k*_cat_/K_M_) for formaldehyde and pyruvate, respectively. Moreover, *K*_i_ for both substrates increased. Variant Q107R showed better turnover and similar *K*_M_ values compared to YfaU-wt, while *K*_i_ for formaldehyde decreased.

The operational stability of the Q107R and Q141L variants in this reaction was evaluated in a batch reactor. Assuming that the decay in operational stability can be described by first order kinetics (Figure S8), the calculated deactivation constants (*k*_d_) for variants Q107R and Q141L are approximately 2-fold lower than for YfaU-wt (Table 4). Both variants showed similar values in terms of operational stability (*k*_d_ and half-life), which contrasts with their differences in kinetic thermostability, where Q107R showed a half-life at 60 °C almost 5-fold higher than Q141L (Table 2). These differences between kinetic and operational stability could be explained by the fact that half-life at high temperature considers only the stability of the protein molecule in buffer, while operational stability considers enzyme activity in the reactor in the presence of substrate, cofactor, and products.^24^

**Table 4:** Estimated Values of Operational Stability Decay Rate Constants (*k*_d_) and the Corresponding Half-Life Times in a Batch Reactor at 25 °C^a^

YfaU variant	*k*_d_ (h^–1^)	half-life (h)
wild-type	0.144 ± 0.013	4.8 ± 0.4
Q107R	0.068 ± 0.006	10.2 ± 0.9
Q141L	0.061 ± 0.006	11 ± 1

a Values represent the mean and the standard deviation of three independent determinations.

### Structure–Function Analysis of YfaU Q107R and Q141L

**Figure 4:**
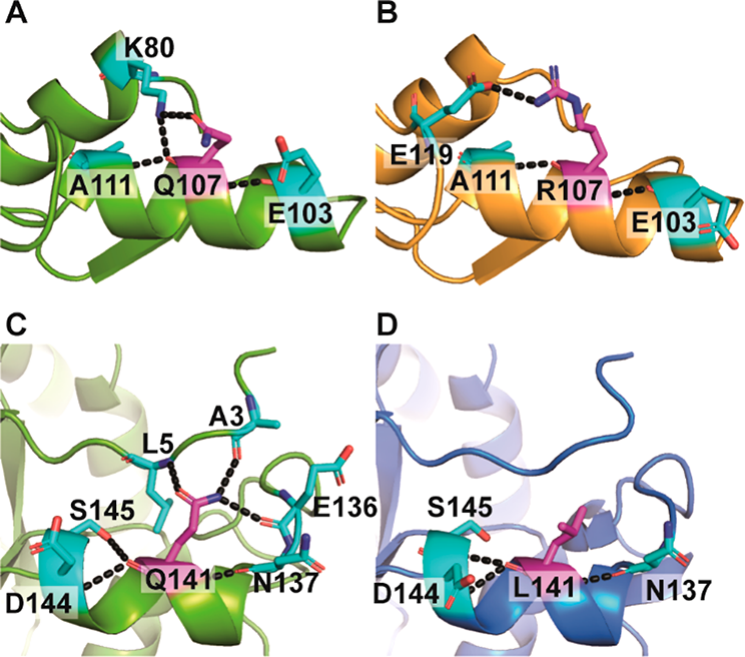
Hydrogen bonds formed between residues 107 and 141 with their surrounding residues, respectively. **A**. Residue Gln107 of YfaU-wt. **B**. Residue Arg107 of variant Q107R. **C**. Residue Gln141 of YfaU-wt. **D**. Residue Leu141 of variant Q141L. Target residues are depicted in magenta sticks in CPK colors, while residues which form hydrogen bonds are illustrated as cyan sticks.

To investigate the reason why both mutants were more thermostable, homology models of Q107R and Q141L were built using the crystal structures of YfaU-wt (PDB: 2VWS and 2VWT). YfaU presents a hexameric assembly composed by a trimer (3-fold axis) of (β/α)_8_ barrel dimers (2-fold axis). Since the 2-fold related subunits superpose with an RMSD of 0.25 Å^25^ and residues Gln107 and Gln141 are not involved in the interaction between subunits, only the 3-fold related subunits were considered for the analysis (Figure S9). The replacement Q107R decreased the number of H-bonds with the replaced residue or with other amino acids in its hydrogen bond network. Similar results were found for the mutant Q141L (Figure 4).

**Figure 5:**
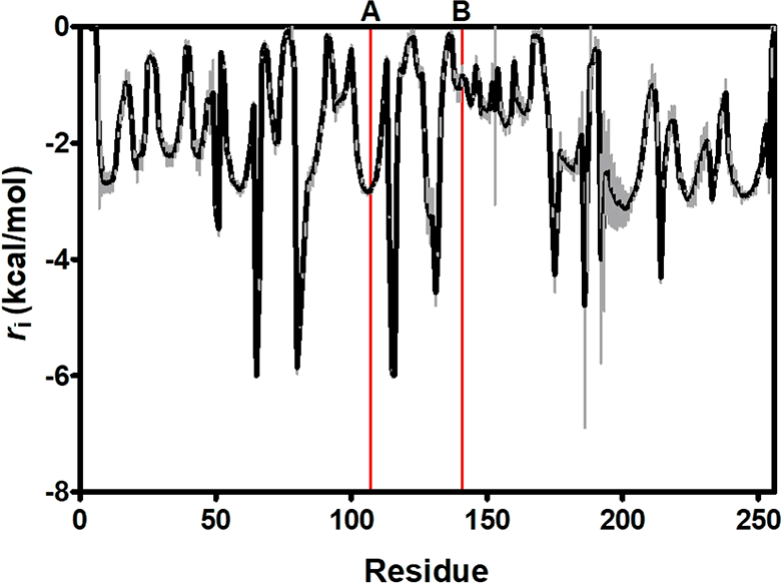
Rigidity index (*r*_i_) for YfaU-wt calculated by CNA. Lines indicate the amino acid replacements (line A corresponds to position 107 and line B, position 141). Since YfaU is a homotrimer, *r*_i_ has been averaged for the same residue of each chain. The mean value is shown in black, and the standard deviations are in gray.

Rigidity index (*r*_i_) from the CNA algorithm was also used to monitor the degree of rigidity of the residues from YfaU-wt (Figure 5). As previously described, only the trimeric assembly was considered for structural analysis. Considering this and since CNA does not relate residues from different chains, *r*_i_ has been averaged from the three different chains. According to CNA, with a *r*_i_ value of −2.8 kcal/mol, residue Gln107 is not in a flexible region of the protein. However, residue Gln141 has a *r*_i_ value of −0.84 kcal/mol, which implies a certain degree of flexibility in this region.

Finally, FoldX calculations were carried out to estimate the folding free energy of the mutants (ΔΔ*G*). Q107R caused a ΔΔ*G* of −2.55 kcal/mol. Considering ΔΔ*G* from FoldX and the general rule that correlates Δ*G*_unfold_ and Δ*T*_m_,^26^ the corresponding empirical Δ*T*_m_ would be 9.2 °C, which is similar to the experimental Δ*T*_m_, 8.9 °C. By contrast, ΔΔ*G* of variant Q141L was 0.08 kcal/mol, which would represent a Δ*T*_m_ of −0.3 °C, while the experimental Δ*T*_m_ was 2.2 °C.

Considering the output of the chosen methods and algorithms used for structure–function analysis, we could identify beneficial mutations using our screening system, which would not be made easily evident by bioinformatics tools. However, the folding interference principle in *T. thermophilus* allowed the identification of these stabilizing positions, in consonance with a recent study in which stabilizing positions were identified in the esterase I from *Pseudomonas fluorescens* also by folding interference, using the kanamycin nucleotidyl tranferase gene as folding reporter instead.^6^

## Conclusions

The improvement of hygromycin B phosphotransferase (Hph17) enabled the thermal stabilization of the pyruvate aldolase from *E. coli* YfaU. The only two selected variants that were expressed in soluble form, YfaU99 and 103, showed higher *T*_m_ than the wild-type protein, 2.0 and 2.9 °C, respectively, at the cost of a lower specific activity. However, the low solubility issue can be solved using complementary rational design strategies, such as specific solubility-enhancing algorithms or back-to-consensus mutations that restore conserved amino acids, which usually yield active and more soluble proteins.^24,26^

With the aim of knowing the effect of individual mutations both in enzyme activity and stability, all mutations were segregated and characterized individually. The Q107R and Q141L replacements conferred higher kinetic and thermodynamic stability. Especially interesting is the case of variant Q107R, with an increase of 8.9 °C in *T*_m_, 16-fold longer half-life, and similar kinetic constants than YfaU-wt. Regarding variant Q141L, the improvement in stability was much more modest, but this variant had better turnover, affinity, and lower substrate inhibition compared to the wild-type.

YfaU is a relevant enzyme for biocatalysis, allowing for instance the synthesis of l-homoserine using alanine and up to 3 M formaldehyde, when coupled with a transaminase.^10^ Our highly active and thermostable Q107R and Q141L variants have twice the operational stability of YfaU-wt in the synthesis of 4-hydroxy-2-oxobutanoate, which would allow a longer-term usage in this cascade reaction, with the consequent reduction in the cost of the process.

## Supporting Information Available

Tables, figures, and experimental procedures (PDF)
