# Supplementary material for: Thermostability Engineering of a Class II Pyruvate Aldolase from Escherichia coli by in Vivo Folding Interference
Source: ACS Sustain Chem Eng. 2021 Apr 7;9(15):5430–6. doi: 10.1021/acssuschemeng.1c00699 (PMC8461973; doi:10.1021/acssuschemeng.1c00699)
Supplement: Supplementary file 1 — sc1c00699_si_001.pdf [file sc1c00699_si_001.pdf]

SUPPORTING INFORMATION

Thermostability engineering of a class II pyruvate aldolase from *Escherichia coli* by *in vivo* folding interference

Sandra Bosch[†]*, Esther Sanchez-Freire*[†]*, María Luisa del Pozo*[†]*, Morana Česnik*[‡]*, Jaime Quesada*[†]*, Diana M. Mate*[†]*, Karel Hernández*[‖]*, Yuyin Qi*[§]*, Pere Clapés*[‖]*, Đurđa Vasić-Rački*[‡]*, Zvjezdana Findrik Blažević*[‡]*, José Berenguer*[†] *and Aurelio Hidalgo*[†]**.*

[†]Department of Molecular Biology, Center of Molecular Biology "Severo Ochoa" (UAM-CSIC), Autonomous University of Madrid, Nicolás Cabrera 1, 28049 Madrid Spain.

[‡]University of Zagreb, Faculty of Chemical Engineering and Technology, Savska c. 16, HR-10000 Zagreb, Croatia.

[‖]Institute of Advanced Chemistry of Catalonia, Biotransformation and Bioactive Molecules Group, Spanish National Research Council (IQAC-CSIC), Jordi Girona 18-26, 08034 Barcelona, Spain.

[§]Prozomix Ltd., Station Court, Haltwhistle, NE49 9HN Northumberland, United Kingdom.

AUTHOR INFORMATION

Corresponding Author

Aurelio Hidalgo: Department of Molecular Biology, Center of Molecular Biology "Severo Ochoa" (UAM-CSIC), Autonomous University of Madrid, Nicolás Cabrera 1, 28049 Madrid Spain. ORCID: http://orcid.org/0000-0001-5740-5584 E-mail for A.H.: ahidalgo@cbm.csic.es

Authors

Sandra Bosch: Department of Molecular Biology, Center of Molecular Biology "Severo Ochoa" (UAM-CSIC), Autonomous University of Madrid, Nicolás Cabrera 1, 28049 Madrid Spain. ORCID: https://orcid.org/0000-0002-8022-2417 E-mail: sbosch@cbm.csic.es

Esther Sánchez-Freire: Department of Molecular Biology, Center of Molecular Biology "Severo Ochoa" (UAM-CSIC), Autonomous University of Madrid, Nicolás Cabrera 1, 28049 Madrid Spain.

María Luisa del Pozo: Department of Molecular Biology, Center of Molecular Biology "Severo Ochoa" (UAM-CSIC), Autonomous University of Madrid, Nicolás Cabrera 1, 28049 Madrid Spain.

Morana Česnik: University of Zagreb, Faculty of Chemical Engineering and Technology, Savska c. 16, HR-10000 Zagreb, Croatia. E-mail: mcesnik@fkit.hr

Jaime Quesada: : Department of Molecular Biology, Center of Molecular Biology "Severo Ochoa" (UAM-CSIC), Autonomous University of Madrid, Nicolás Cabrera 1, 28049 Madrid Spain.

Diana M. Mate: Department of Molecular Biology, Center of Molecular Biology "Severo Ochoa" (UAM-CSIC), Autonomous University of Madrid, Nicolás Cabrera 1, 28049 Madrid Spain. ORCID: https://orcid.org/0000-0002-8209-0542 E-mail: diana.mate@cbm.csic.es

Karel Hernández: Institute of Advanced Chemistry of Catalonia, Biotransformation and Bioactive Molecules Group, Spanish National Research Council (IQAC-CSIC), Jordi Girona 18-26, 08034 Barcelona, Spain. E-mail: khsqbm@cid.csic.es

Yuyin Qi: Prozomix Ltd., Station Court, Haltwhistle, NE49 9HN Northumberland, United Kingdom. E-mail: yuyin.qi@prozomix.com

Pere Clapés: Institute of Advanced Chemistry of Catalonia, Biotransformation and Bioactive Molecules Group, Spanish National Research Council (IQAC-CSIC), Jordi Girona 18-26, 08034 Barcelona, Spain. ORCID: https://orcid.org/0000-0001-5541-4794 E.mail: pere.clapes@iqac.csic.es

Đurđa Vasić-Rački: University of Zagreb, Faculty of Chemical Engineering and Technology, Savska c. 16, HR-10000 Zagreb, Croatia. E-mail: dvracki@fkit.hr

Zvjezdana Findrik Blažević: University of Zagreb, Faculty of Chemical Engineering and Technology, Savska c. 16, HR-10000 Zagreb, Croatia. ORCID: https://orcid.org/0000-0002-5312-8951 E-mail: zfindrik@fkit.hr

José Berenguer: Department of Molecular Biology, Center of Molecular Biology "Severo Ochoa" (UAM-CSIC), Autonomous University of Madrid, Nicolás Cabrera 1, 28049 Madrid Spain. ORCID: http://orcid.org/0000-0002-9689-6272 E-mail: jberenguer@cbm.csic.es

CONTENTS

1. **Experimental Procedures (pages S4-S13)**

2. **Supporting Figures (pages S14-S23)**

Figure S1. Selection method based on the folding interference principle.. ....S14

Figure S2. Selection conditions for plasmid pMH184 in *T. thermophilus*. S15

Figure S3. Secondary screening of selected clones from randomized libraries of Hph5 at (**A**) 71 and (**B**) 74 °C using a decimal dilution assay.. ....S16

Figure S4. Selection conditions for plasmid pNCH-YfaUwt in *T. thermophilus*. S17

Figure S5. Secondary screening of selected clones from a randomized YfaU library at 67 °C using a decimal dilution assay. S18

Figure S6. SDS-PAGE gels (12% acrylamide) showing lysates of putative thermostable YfaU variants obtained by folding interference principle. S20

Figure S7. Kinetics of aldol addition of pyruvate and formaldehyde catalysed by Yfau-wt, Q107R and Q141L (50 mM sodium phosphate buffer pH 7.0, γ_{Yfau} = 1 mg/mL)..S21

Figure S8. Change of relative activity of YfaUs during the aldol addition of formaldehyde to pyruvate...S22

Figure S9. Cartoon representation of YfaU-wt structure..S23

3. **Supporting Tables (pages S24-S26)**

Table S1. Primers used to sequence, amplify or subclone *hph* or *yfaU* genes. S24

Table S2. Primers used to insert individual mutations into the *hph* gene. S25

Table S3. Primers used to insert individual mutations into the *yfaU* gene. S26

1. EXPERIMENTAL PROCEDURES

Strains and growth media

E. coli DH5α [*sup*E44, □*lac*U169 (□80 *lac*Z□M15), *hsd*R17, *rec*A, *end*A1, *gyr*A96, *thi-1, relA1*] was used for molecular cloning and *E. coli* BL21(□DE3) [*hsd*S, *gal* (□*c*Its857, *ind*1, *Sam7, nin5, lacUV5*-T7 gene 1)] was used for protein production. Both strains were grown in Luria-Bertani lysogeny broth (LB; 10 g/L tryptone, 10 g/L NaCl and 5 g/L yeast extract). *T. thermophilus* HB27 was grown in *Thermus* broth (TB; 8 g/L tryptone, 4 g/L yeast extract and 3 g/L NaCl in carbonate-rich mineral water).[9] Media were solidified by addition of 1.5% (w/v) of agar, if required. After autoclaving and upon cooling down, media were supplemented with a final concentration of 100 μg/mL of ampicillin, 30 μg/mL of kanamycin, or 100 μg/mL of hygromycin B (HygB), where required.

E. coli was grown at 37 °C for 12 h. *T. thermophilus* was grown at 60-74 °C for 24-48 h. Liquid cultures were shaken in an orbital incubator at 180 rpm at their corresponding temperature. To verify the temperature of the heater an iButton Thermo-S-kit-T thermochron Starter Kit 0-125 °C (Measurement Systems LTD) was used.

Library construction and selection of Hph variants

A randomized library of the moderately thermostable HygB phosphotransferase, *hph5,* was obtained by error-prone PCR (epPCR)[10] using the *E. coli-T. thermophilus* shuttle plasmid pMH184[11] as template, 0.05 U/μl NZYtaq DNA polymerase, 0.5 μM of each primer ep_HPH_fw and ep_HPH_rv (**Table S1**) and 0.2 mM $MnCl_2$. The corresponding epPCR products were digested with NdeI and BglII, cloned in plasmid pMH184[11] replacing the wild-type *hph* gene, transformed in *E. coli* DH5α, pooled and their DNA extracted. In this *Thermus-E. coli* shuttle plasmid, the *hph5* variants were expressed

constitutively under the control of the S-layer gene promoter of *T. thermophilus* (*PslpA*), also active in *E. coli*. Primer hph_sec1 was used for sequencing variants of *hph5*.

The resulting pMH184-epPCR*hph5* was transformed in *T. thermophilus* HB27 by natural competence by adding 100 ng of plasmid DNA to 0.5 mL of cell culture at exponential phase (OD_{600} 0.3-0.4) in TB medium. Cultures were incubated 4 h after the addition of DNA and the suspension was directly spread on selection plates with antibiotic or diluted before plating for viability assays.

Hph5 as folding reporter

The *E. coli*-*T. thermophilus* folding intereference vector pNCH was constructed from pNCK.[5] The gene that confers resistance to kanamycin was replaced by the parent type *hph5* or the evolved variant *hph17.* These genes were cloned using the primers Hph_Notlinker_fw and Hph_Eco_TTA_rv (**Table S1**) to include the restriction sides NotI and EcoRI, respectively. The primer Hph_Notlinker_fw also contains the nucleotides encoding the amino acid residues AAAGSSGSI which constitutes the linker between the protein of interest and either Hph5 or Hph17.

The resulting plasmids were transformed into *T. thermophilus* HB27 by natural competence as described above.

Library construction and selection of YfaU variants

A randomized library of the *E. coli* class II pyruvate aldolase, YfaU (UniProt: P76469), was obtained similarly. The $MnCl_2$ concentration used for the epPCR was 0.3 mM and the primers used were ep_pNCK_fw and ep_pNCH_rv (Table S1). The resulting epPCR products were digested with NcoI and NotI and ligated into pNCH. Primer pslpA_fw was used for sequencing YfaU variants (**Table S1**).

The resulting pNCH-epPCR*yfaU* was transformed in *T. thermophilus* HB27 and spread on selection plates for viability assays as explained above.

Site-directed mutagenesis

The generation of Hph17 derivatives with single substitutions (R61H, S86G, Q96P, A185V or V322E) and YfaU (L4F, G90S, Q107R, V122F, Q141L, P187T, F215L, A252E, F254I, P261Q and I263K) was performed using the QuikChange site-directed mutagenesis kit (Agilent Genomics), following the manufacturer's protocol and primers listed in **Tables S2** and **S3**, respectively.

Protein expression and purification

To subclone *hph5*, *hph17* and their derivatives into plasmid pET28b for recombinant expression, primers hph_fw_Nde and hph_rv_Eco were designed with the restriction sites NdeI and EcoRI, respectively (**Table S1**). The selected YfaU variants and their derivatives were also cloned into plasmid pET28b, the encoding genes were amplified by PCR using primers YfaU_Nde_fw and YfaU_Hind_rv (**Table S1**) with the restriction sites NdeI and HindIII, respectively. In both cases, pET28b allowed overexpression of the proteins in *E. coli* BL21 (DE3) as His-tagged products under the control of T7 RNA polymerase promoter.

Chemically competent *E. coli* DH5α cells were transformed using 5 µL of ligation or 100-200 ng of plasmid.[12] Electroporation of *E. coli* BL21 (DE3) was carried out by mixing 45 µL of competent cells with 100-200 ng of plasmid and subjecting the cells to a short pulse (5 ms) at 12500 V/cm (EasyjectPlus D2000; 2500 V, 201 Ω and 25 µF), using 0.2 cm gap cuvettes (Bio-Rad). Immediately after the pulse, 500 µL of SOB medium were added and incubated for 1 h before plating on selective solid medium.

The generated pET28b-*hph* and pET28b-*yfaU* variant-harboring constructs were transformed in *E. coli* BL21. One colony of each insert was grown overnight in LB liquid with kanamycin. Then, 1 mL of culture medium was inoculated in 100 mL of lactose autoinduction medium[13] and incubated overnight at 20°C and 180 rpm for protein expression. The induced cultures were centrifuged and re-suspended in 30 mL of Buffer A (50 mM sodium phosphate buffer pH 7.4, 500 mM NaCl supplemented with 20 mM imidazole) for Hph variants or in Buffer B (50 mM sodium phosphate buffer pH 8, 300 mM NaCl supplemented with and 20 mM imidazole) for YfaU variants. Cells were lysed with a pressure homogenizer *Niro SOavi* (GEA). Cellular debris was removed by centrifugation at 15,000 ×g for 30 min. The cell extract was mixed with 1 mL of Ni-NTA resin (Ni-NTA Superflow, Qiagen), incubated at 4°C in a shaker for 60 min and packed in a plastic column. The column was washed with Buffer A or Buffer B, respectively. Lastly, bound proteins were recovered with elution buffer for Hph variants (Buffer A with 500 mM imidazole) or with elution buffer for YfaU (Buffer B with 500 mM imidazole). The purified Hph variants were concentrated using Amicon Ultra-15 concentrators with a 10-kDa cutoff (Merck Millipore). The buffer of the purified YfaU variants was exchanged with storage buffer (10 mM sodium phosphate buffer pH 7 and 100 mM NaCl) using Spectra/Por membrane tubing with a cutoff of 6-8 kDa (Spectrum Labs).

After protein concentration, purity was checked by SDS-PAGE (Sodium Dodecyl Sulfate Polyacrylamide Gel Electrophoresis) in a 12% polyacrylamide gel using the Bio-Rad low-range SDS-PAGE protein standard. Protein concentrations were determined using the Bio-Rad Protein Assay (Bio-Rad) according to the manufacturer's protocol, using bovine serum albumin (BSA) as standard.

The gene encoding pyruvate kinase (PK) from *T. thermophilus* HB27 (Uniprot ID: Q72H84) was cloned from *T. thermophilus* HB27 genomic DNA using the primers TtPK_Nde_fw and TtPK_Hind_rv (**Table S1**) with the restriction sites NdeI and HindIII, respectively. The resulting PCR product was subcloned into pET22b using T4 DNA ligase (Promega). The gene encoding lactate dehydrogenase (LDH) from *Thermotoga maritima* (UniProt ID: P16115), was purchased from Thermo Fisher Scientific including NdeI and EcoRI restriction sites for cloning and optimizing their codons for expression in *E. coli*. After double digestion with NdeI and EcoRI, the target gene was subcloned into pET28b applying T4 DNA ligase. Both genes were expressed and the corresponding proteins were purified using the same protocol as the purification of Hph, described above.

Activity assays for Hph variants

Hph activity was determined as the decrease of NADH[14] at 60°C using a coupled assay with PK from *T. thermophilus* HB27 and LDH from *T. maritima* as auxiliary enzymes. Briefly, 0.5 µg of pure Hph protein was incubated with 1 U/mL of each auxiliary enzyme in 50 mM Tris-HCl, 10 mM $MgCl_2$, 20 mM KCl and 40 mM $MgSO_4$, pH 7.5, 0-5 mM HygB, 0-5 mM ATP, 5 mM phosphoenolpyruvate and 1 mM NADH in a Fluostar optima plate reader (BMG Labtech), and the decrease in absorbance at 340 nm recorded for 15 min. The reaction was started by addition of Hph and HygB (in that order) to a preheated reaction mix containing the rest of the components. Assays were carried out in triplicate. One unit of activity was defined as the amount of enzyme that consumes 1 µmol of ATP per min under the assay conditions.

Activity assays for YfaU variants

YfaU retro-aldol cleavage of 4-hydroxy-2-oxopentanoate to pyruvate and acetaldehyde coupled with alcohol dehydrogenase at 25°C was used to determine YfaU activity. For this assay, 0.5 µg of pure YfaU protein was incubated with 10 U/mL of *Saccharomyces cerevisiae* alcohol dehydrogenase (Sigma) as auxiliary enzyme in 50 mM sodium phosphate buffer, pH 7.5, 4 mM of substrate (4-hydroxy-2-oxopentanoate) and 1 mM NADH in a Fluostar optima plate reader (BMG Labtech) and the decrease in absorbance at 340 nm recorded for 20 min. The reaction was started by addition of substrate to a reaction mix containing the rest of the components. Assays were carried out in triplicate. One unit of activity was defined as the amount of enzyme that consumes 1 µmol of 4-hydroxy-2-oxopentanoate per min under the assay conditions.

Stability assays

The thermodynamic stability of the different Hph or YfaU variants was determined using differential scanning fluorimetry (DSF).[15] A 20 µM solution of each protein in PBS for Hph variants (137 mM NaCl, 2.7 mM KCl, 8.1 mM Na_2HPO_4 and 1.5 mM KH_2PO_4) or 10 mM sodium phosphate buffer pH 7.0 and 100 mM NaCl for YfaU variants was supplemented with 5x SYPRO Orange (Sigma-Aldrich) and subjected to a temperature ramp from 35 to 95 °C at 1 °C/min in a Rotor Gene™ 6000 (Corbett Life Sciences) real time thermocycler. The assay was carried out in triplicate.

The kinetic stability of the different Hph variants was determined by incubating solutions of 1.2 µM of the Hph variants in PBS at 65 °C and determining the residual activity over time using the coupled assay described above. The kinetic stability at 60 °C was also measured for the different YfaU variants in 50 mM sodium phosphate buffer pH 7.0 and 100 mM NaCl to achieve a final concentration of 0.1 mg/mL. The resulting curves

were modeled to first-order deactivation kinetics to calculate the deactivation constants and half-lives.

Steady-state kinetic parameter determination and modeling of YfaU variants

Pyruvate aldolase activities were measured by using the initial reaction rate method in 0.5-mL batch reactor in 50 mM sodium phosphate buffer pH 7.0 and at 25 °C. The linear slope, estimated from the change of product (4-hydroxy-2-oxobutanoate) concentration at the beginning of the reaction when substrate conversion was <10%, was used to calculate the specific enzyme activity (Equation 1). The change in product concentration was monitored by HPLC.

$$Specific\ activity\ \left(\frac{U}{mg}\right) = \frac{dc_{product}}{dt} \cdot \frac{V_{reaction}}{V_{enzyme}} \cdot \frac{1}{\gamma_{enzyme}} \qquad \textbf{(Equation 1)}$$

The influence of concentration of each substrate (formaldehyde and pyruvate) on the initial reaction rate, i.e. on the enzyme specific activity, was examined by keeping constant the concentration of the other substrate. The concentration of each substrate was varied up to ca. 800 mM. A series of batch reactor experiments were carried out for that purpose.

Kinetic parameters were estimated by using the software SCIENTIST from the gathered experimental data in the form of dependence of the initial reaction rate on the concentration of substrate and product by applying double-substrate Michaelis-Menten kinetics.

Enzyme mass concentration (γ) differed for each tested YfaU due to different initial reaction rates and is included in specific enzyme activity calculation (**Equation 1**). Mass concentrations for YfaU-wt, Q107R and Q141L were 1.0, 1.0 and 0.3 mg/mL, respectively.

The reaction was monitored by HPLC (Prominence, Shimadzu, Japan) with UV detection at 215 nm. Derivatization with $BnONH_2·HCl$ was required before analysis of substrates (formaldehyde and pyruvate) and product (4-hydroxy-2-oxobutanoate). Phenomenex Kinetex Core-Shell Technology C18 column, 5 μm, 4.6 × 250 mm was used. Solvent A contained 0.1% (v/v) trifluoroacetic acid (TFA) in H_2O, and solvent B 0.095% (v/v) TFA in CH_3CN/H_2O 4:1. The flow rate was 1.5 mL/min, the detection was measured at 215 nm and column temperature was 30 °C. The gradient elution was 10 to 100% B over 10 min, then 2 min at 100% B, and finally from 100 to 10% B for 3 min and 10% B for 1 min. With this column, concentration linearity range for 4-hydroxy-2-oxobutanoate, pyruvate and formaldehyde were 10, 30 and 40 mM, respectively. Thus, this had to be taken into consideration for sample dilution purposes. Retention times for 4-hydroxy-2-oxobutanoate, pyruvate and formaldehyde were 6.1, 7.6 and 8.3 min, respectively.

Operational stability of YfaU variants

YfaU wt, YfaU 3 and Yfau 5 (0.125 mg mL^{-1} of pure enzymes) were compared according to their operational stability in the aldol addition of pyruvate (100 mM) to formaldehyde (100 mM) through ca 24 hours. The reaction was carried out in 50 mM phosphate buffer pH 7.0 at 25 °C. The overall working volume was 2 mL, and during the reaction 5 samples of 40 μL were taken to determine the enzyme activity. Before the measurement of YfaU activity, sample containing the enzyme was filtered by centrifugation using an Amicon filter unit (cut-off 10 kDa) and washed with buffer to remove the substrate and re-suspended in 100 μL of buffer. The reaction was monitored by HPLC as explained above. The concentration of YfaU in the activity assay was set to 0.02 mg/mL. Activities of YfaUs were measured during the aldol addition of formaldehyde (100 mM) to pyruvate (100 mM) using the initial reaction rate method in

0.25-mL batch reactor and in 50 mM phosphate buffer pH 7.0 at 25 °C. The linear slope-estimated from the change in the product concentration at the beginning of the reaction when substrate conversion was <10% was used to calculate specific enzyme activity (**Equation 1**).

Structural modeling of Hph and YfaU variants

Structural models of the Hph and YfaU variants were created using the program YASARA Structure (YASARA Biosciences) using the default automated macro.[16] The templates for Hph variants were selected automatically (PDB codes 3W0O, 3W0P, 3W0Q, 3W0R and 3W0S), while the templates selected to model YfaU variants were those with PDB code 2VWS and 2VWT. Loops were modelled using a maximum of 50 conformations per loop. The best parts of the models were combined to obtain a hybrid model. Quality assessment images were generated with PyMol v1.3 (The PyMOL Molecular Graphics System, Version 1.3, Schrödinger, LLC.).

Bioinformatic analysis of gene sequences

The assembly, alignment, and translation of gene sequences were carried out using the SnapGene software.

Bioinformatic analysis of enzyme stability

Molecular dynamics (MD) simulations were performed with the YASARA software with the AMBER14 force field. The starting wild-type YfaU was obtained from PDB 2VWS and 2VWT and the structure of variants Q107R and Q141L was obtained by homology modeling using YASARA as described above. Periodic boundary conditions were applied and the energy of the system was minimized. The MD was carried out with

the default automated macro. The simulations were run at 25ºC and 200ºC for 20 ns with a time step of 100 ps. RMSD and RMSF values of the alpha carbon of each residue were calculated analyzing the trajectories for the entire MD disregarding the first 2 ns of equilibration.

The constraint network analysis (CNA) was carried out using the rigidity analysis based on a single network. The number of hydrophobic contacts remained constant during the simulation.[17] All the analyses were done using the CNA web server (http://cpclab.uni-duesseldorf.de/cna).

2. SUPPORTING FIGURES

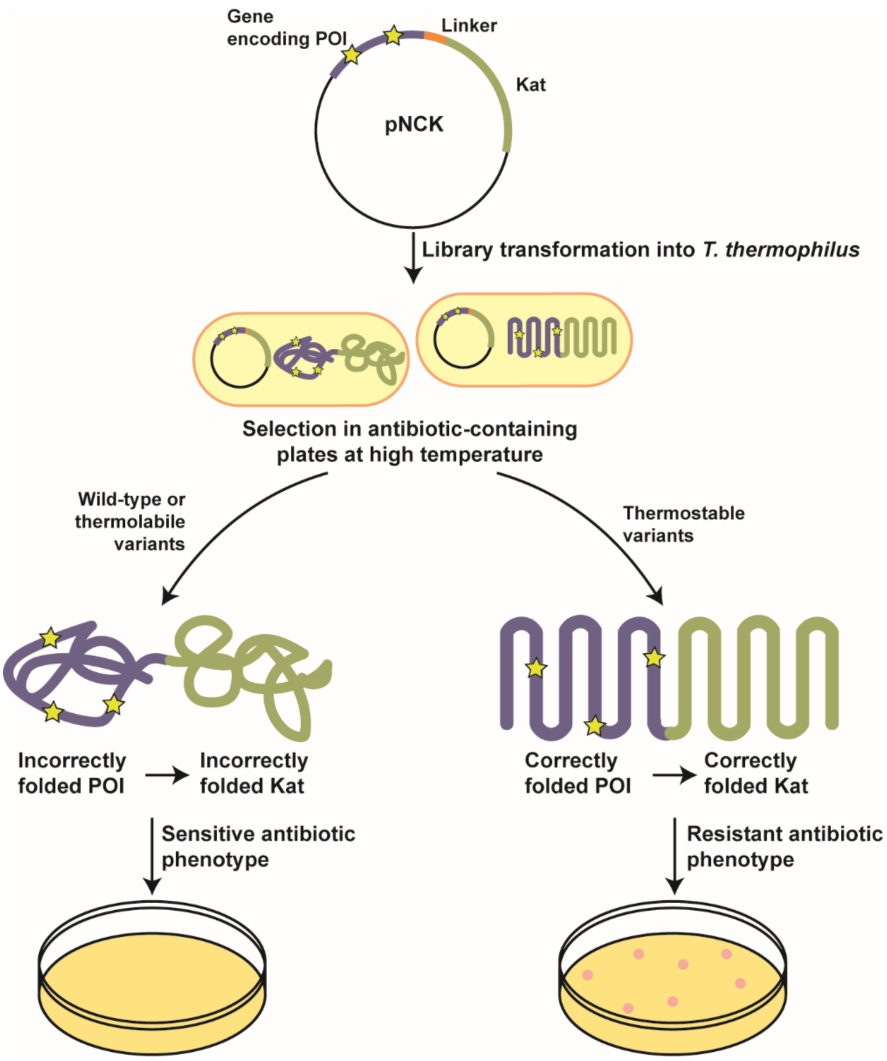

Figure S1. Selection method based on the folding interference principle. A library of the protein of interest (POI) is cloned into a plasmid that enables the fusion at the C-terminus of an antibiotic resistance reporter, Kat in this case, and transformed into *T. thermophilus*. By applying selection pressure at high temperature and in the presence of antibiotic, thermolabile variants will not fold correctly, impeding the proper folding of the antibiotic reporter, and thus, yielding sensitive hosts. On the contrary, thermostable variants will fold correctly allowing, in turn, the correct folding of Kat and thus, stable variants will be identified by growth of colonies.

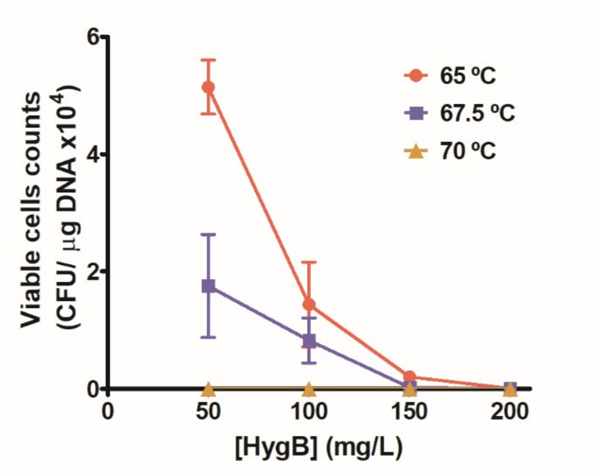

Figure S2. Selection conditions for plasmid pMH184 in *T. thermophilus*. *T. thermophilus* HB27 was transformed with 0.1 µg of DNA by natural competence. Values are the mean of three independent transformations, and error bars represent the standard deviation of the mean.

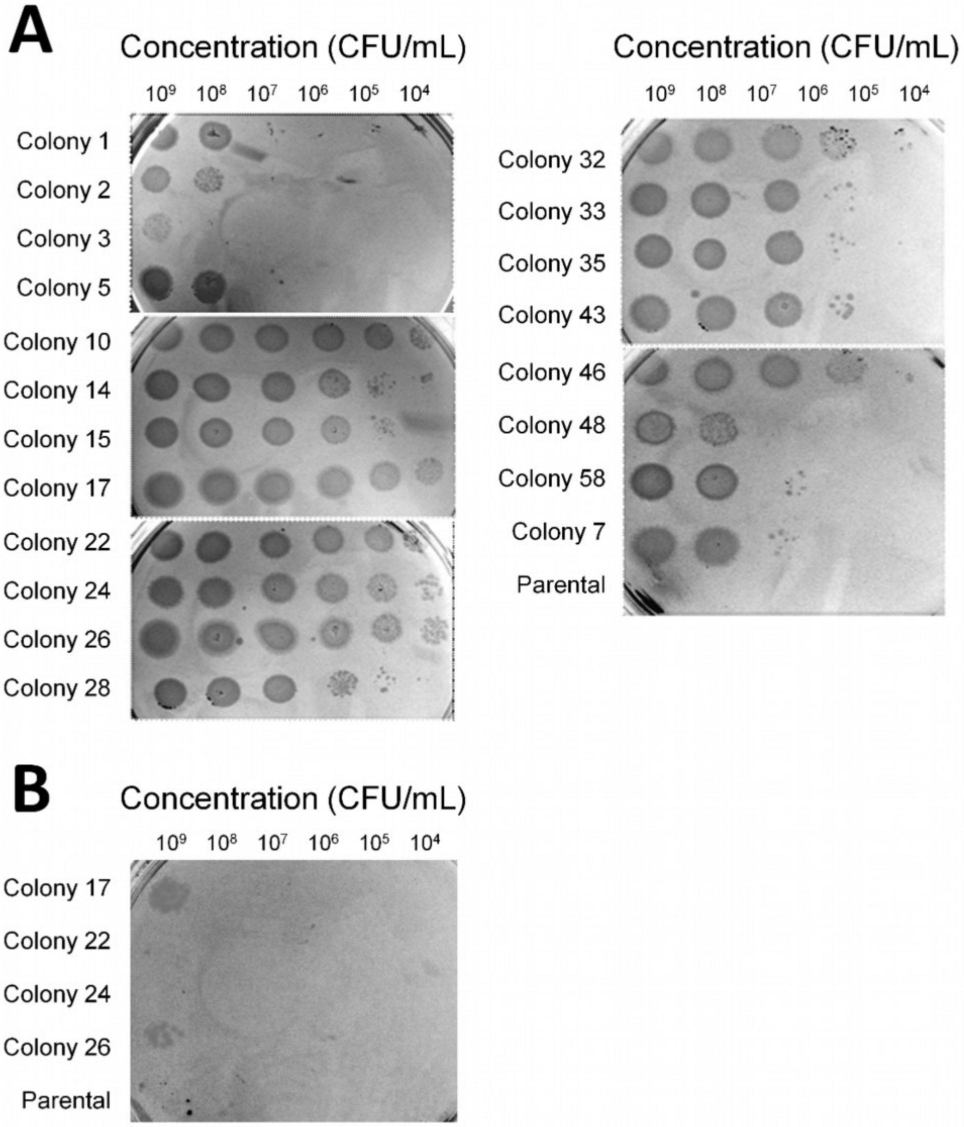

Figure S3. Secondary screening of selected clones from randomized libraries of Hph5 at (**A**) 71 and (**B**) 74 °C using a decimal dilution assay. *T. thermophilus* HB27 was transformed with 0.1 µg of DNA and subsequently grown for 4 h at 60 °C. Ten microliter of different decimal dilutions were spotted on TB plates supplemented with 100 µg/mL HygB and grown at the respective temperature for 48 h. The experiment was carried out in triplicate and a representative image is shown.

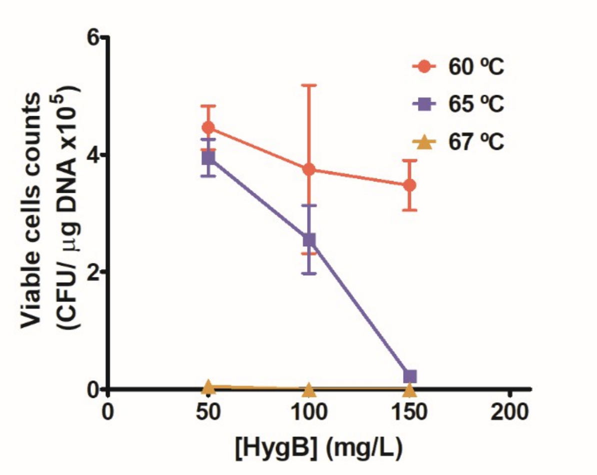

Figure 4. Selection conditions for plasmid pNCH-YfaUwt in *T. thermophilus*. *T. thermophilus* HB27 was transformed with 0.1 µg of DNA by natural competence. Values are the mean of three independent transformations, and error bars represent the standard deviation of the mean.

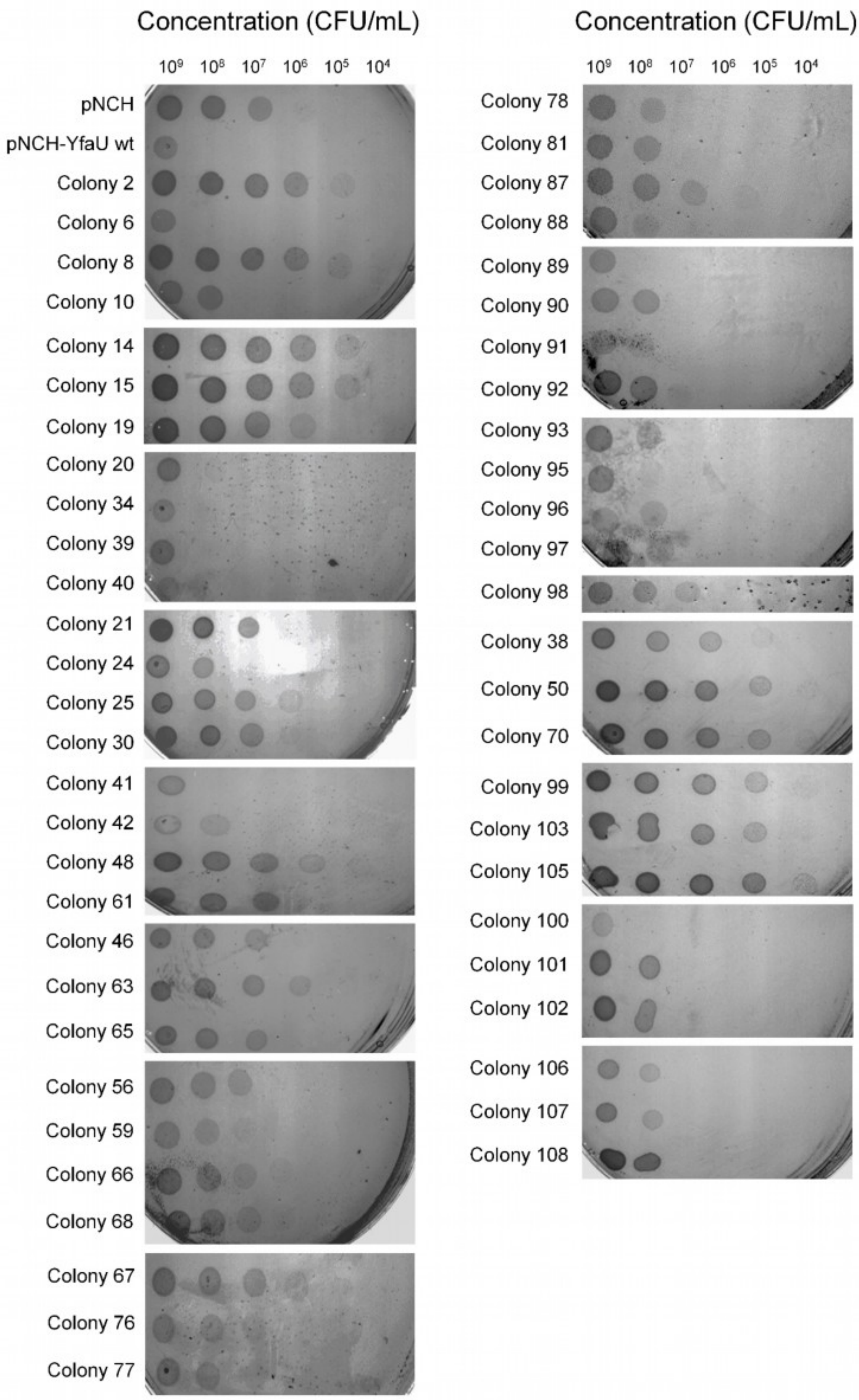

Figure S5. Secondary screening of selected clones from a randomized YfaU library at 67 °C using a decimal dilution assay. *T. thermophilus* HB27 was transformed with 0.1 µg

of DNA and subsequently grown for 4 h at 60 °C. Ten microliter of different decimal dilutions were spotted on TB plates supplemented with 100 µg/mL HygB and grown at 67 °C for 48 h. The experiment was carried out in triplicate and a representative image is shown.

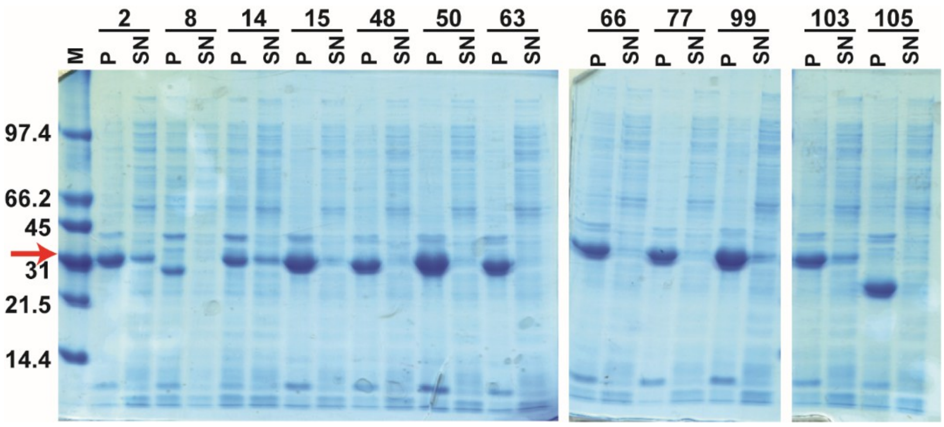

Figure S6. SDS-PAGE gels (12% acrylamide) showing lysates of putative thermostable YfaU variants obtained by folding interference principle. The solubility of each variant was checked by separating supernatant (SN) and pellet (P) from the lysates. Pure: purified YfaU wild-type. M: protein markers with 14.4, 21.5, 31, 45, 66.2 and 97.4 kDa (Bio-Rad).

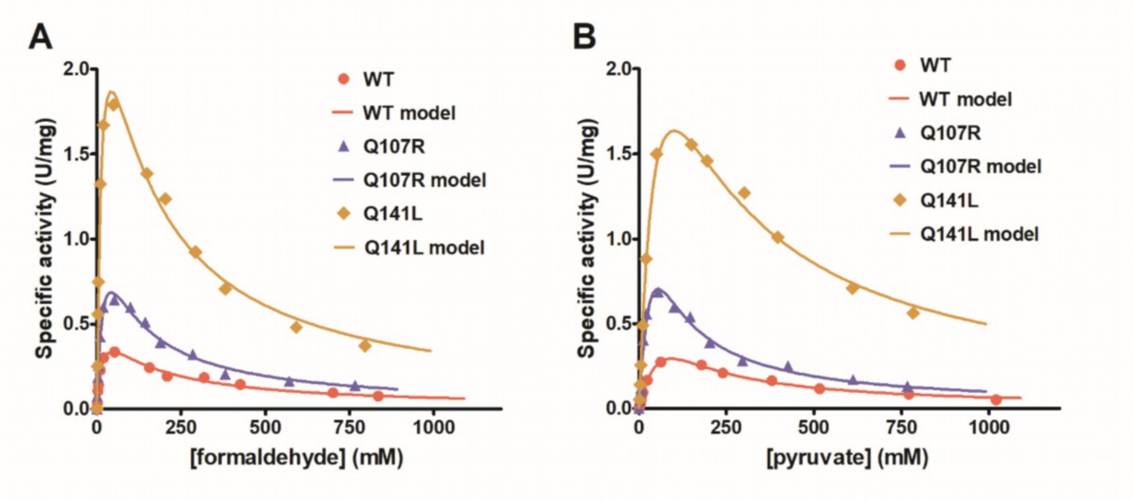

Figure S7. Kinetics of aldol addition of pyruvate to formaldehyde catalysed by YfaU-wt, Q107R and Q141L (50 mM sodium phosphate buffer pH 7.0, γ_{YfaU} = 1 mg/mL). **A.** Pyruvate concentration = 100 mM, **B.** Formaldehyde concentration = 100 mM.

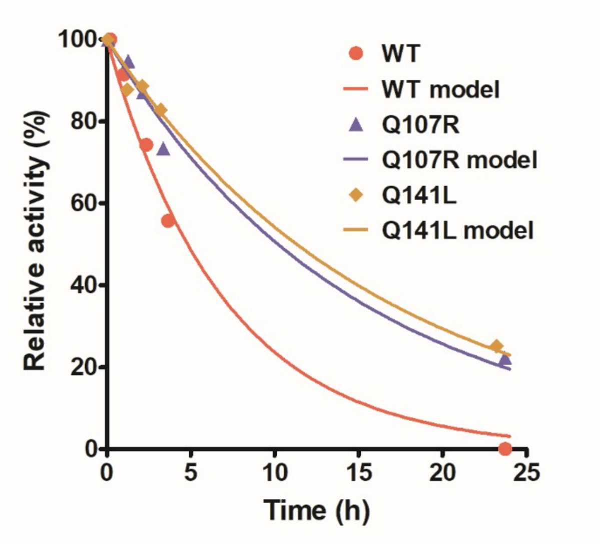

Figure S8. Change of relative activity of YfaUs during the aldol addition of formaldehyde to pyruvate (50 mM phosphate buffer pH 7.0, 25 °C, V_{reactor} = 2 mL, $c_{\text{formaldehyde}}$ = c_{pruvate} = 100 mM, ρ_{YfaU} = 0.125 mg/mL).

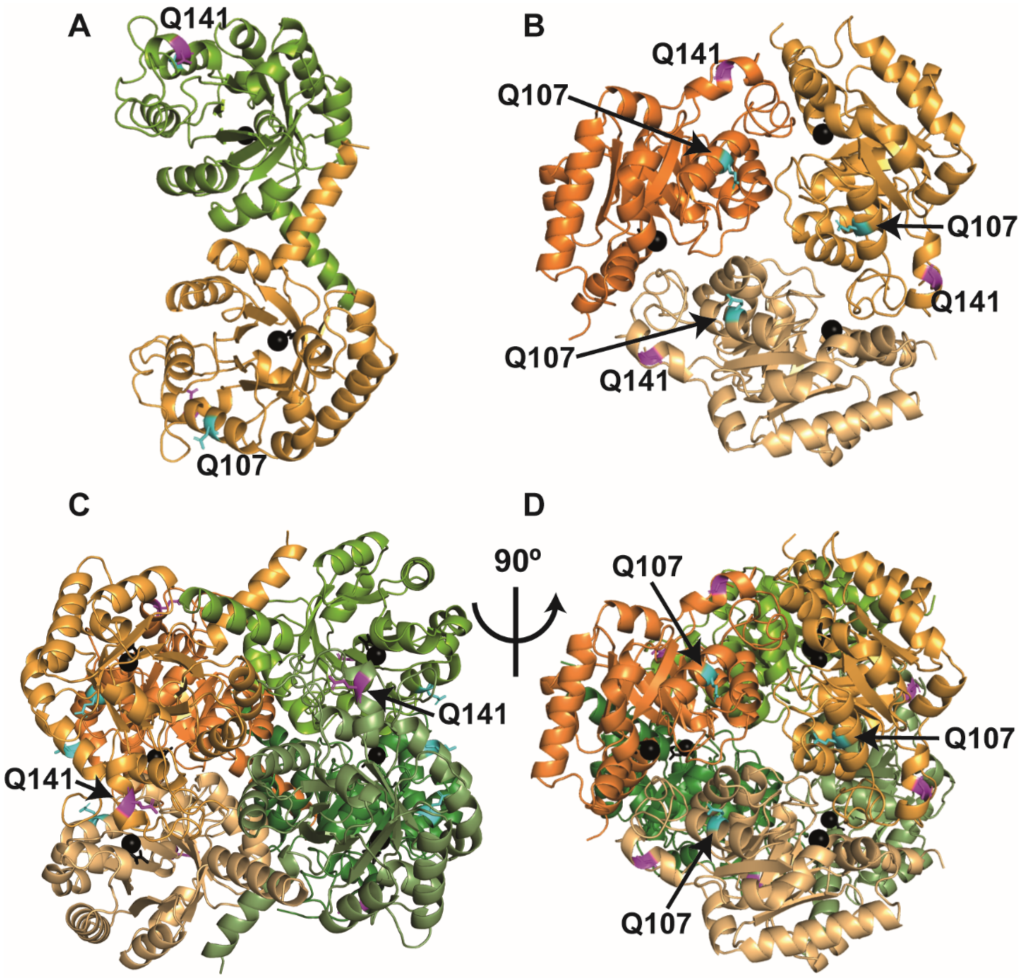

Figure S9. Cartoon representation of YfaU-wt structure. (A) Two (β/α)8 barrels form a domain-swapped dimer (2-fold symmetry axis). Monomers are depicted in orange and green, respectively. (B) The three subunits forming the 3-fold symmetry axis, each monomer is illustrated in different shades of orange. (C) and (D) Three domain-swapped dimers forming the hexameric assembly, (C) front view, (D) side view, which is rotated 90°. The Mg^{2+} ions and the pyruvate molecules are illustrated in black. Substituted residues Q107 and Q141 can be localized as cyan and magenta sticks, respectively.

3. SUPPORTING TABLES

Table S1. Primers used to sequence, amplify or subclone *hph* or *yfaU* genes. Restriction sites are underlined.

Name	Sequence (5'→ 3')
**ep_HPH_fw**	GCCCACGACCCTTAAGGA
**ep_HPH_rv**	CTGGCACAGATGGTCATAACC
**hph_sec1**	CGGAGAGGAAACGCGGCAAC
**Hph_Notlinker_fw**	AAAAAAGCGGCCGCAGGAAGCTCTGGTTCCATCAAAAAGCCTGAACTCA
**Hph_Eco_TTA_rv**	AAAAAAGAATTCTTATTCCTTTGCCCTCGGACG
**ep_pNCK_fw**	CCCACGACCCTTAAGGAGGT
**ep_pNCH_rv**	GAGTTCAGGCTTTTTGATGGAACCAGAG
**pslpA_fw**	CCCGGGAGTATAACAGAAACC
**hph_fw_Nde**	AAAAAACATATGAAAAAGCCTGAACTCACCG
**hph_rv_Eco**	AAAAAAGAATTCCTATTCCTTTGCCCTCGGAC
**YfaU_Nde_fw**	AAAAAACATATGAACGCATTATTAAGC
**YfaU_Hind_rv**	AAAAAAAAGCTTAATAACTACCTTTTATG
**TtPK_Nde_fw**	TTTTTTCATATGCCGCCTTTTAAGCG
**TtPK_Hind_rv**	TTTTTAAGCTTCCCCACCCGCTCCA

Table S2. Primers used to insert individual mutations into the *hph* gene. The mutated codon is underlined.

**Name**	**Sequence 5’ → 3’**
**QX_hph5_R61H_fw**	CGATGGTTTCTACAAAGATCATTATGTTTATCGGCACTTTGC
**QX_hph5_R61H_rv**	GCAAAGTGCCGATAAACATAATGATCTTTGTAGAAACCATCG
**QX_hph5_S86G_fw**	TTGGGGAATTTAGCGAGGGCCTGACCTATTGCATC
**QX_hph5_S86G_rv**	GATGCAATAGGTCAGGCCCTCGCTAAATTCCCCAA
**QX_hph5_Q96P_fw**	CCCGCCGTGCACCGGGTGTCACGTT
**QX_hph5_Q96P_rv**	AACGTGACACCCGGTGCACGGCGGG
**QX_hph5_A185V_fw**	CTGATGCTTTGGGTCGAGGACTGCCCC
**QX_hph5_A185V_rv**	GGGGCAGTCCTCGACCCAAAGCATCAG
**QX_hph5_V322E_fw**	GACCGATGGCTGTGAAGAAGTACTCGCCG
**QX_hph5_V322E_rv**	CGGCGAGTACTTCTTCACAGCCATCGGTC

Table S3. Primers used to insert individual mutations into the *yfaU* gene. The mutated codon is underlined.

**Name**	**Sequence (5'->3')**
**QX_YfaU_L5F_fw**	GGCAGCCATATGAACGCATTTTTAAGCAATCCCTTTAAAGA
**QX_YfaU_L5F_rv**	TCTTTAAAGGGATTGCTTAAAAATGCGTTCATATGGCTGCC
**QX_YfaU_G90S_fw**	GCTGATTAAACAAGTCCTGGATATTAGCGCGCAAACTC
**QX_YfaU_G90S_rv**	AGAGTTTGCGCGCTAATATCCAGGACTTGTTTAATCAGC
**QX_YfaU_Q107R_fw**	CCGAACAGGCACGTCGTGTGGTGTCTGCCACG
**QX_YfaU_Q107R_rv**	CGTGGCAGACACCACACGACGTGCCTGTTCGG
**QX_YfaU_V122F_fw**	CCTACGGTGAGCGTGGTTTTGGGGCCAGTGT
**QX_YfaU_V122F_rv**	ACACTGGCCCCAAAACCACGCTCACCGTAGG
**QX_YfaU_Q141L_fw**	GCATTGAGAATTACATGGCGCTGGTTAACGATTCGCTTTGTCTG
**QX_YfaU_Q141L_rv**	CAGACAAAGCGAATCGTTAACCAGCGCCATGTAATTCTCAATGC
**QX_YfaU_P187T_fw**	GTCGTTGGGCTACACCGATAACGCCGGGC
**QX_YfaU_P187T_rv**	GCCCGGCGTTATCGGTGTAGCCCAACGAC
**QX_YfaU_F215L_fw**	GGTAAAGCGGCTGGTCTGCTGGCTGTGGCTCCT
**QX_YfaU_F215L_rv**	AGGAGCCACAGCCAGCAGACCAGCCGCTTTACC
**QX_YfaU_A252E_fw**	GATGCCCTGGATCAACGACTGGAAATGTTTAAATCAGGCAAAAATGG
**QX_YfaU_A252E_rv**	CCATTTTTGCCTGATTTAAACATTTCCAGTCGTTGATCCAGGGCATC
**QX_YfaU_F254I_fw**	GGATCAACGACTGGCGATGATTAAATCAGGCAAAAATGG
**QX_YfaU_F254I_rv**	CCATTTTTGCCTGATTTAATCATCGCCAGTCGTTGATCC
**QX_YfaU_P261Q_fw 2**	ATGTTTAAATCAGGCAAAAATGGGCAGCGCATAAAAGGTAGTTATTAAGCTT
**QX_YfaU_P261Q_rv 2**	AAGCTTAATAACTACCTTTTATGCGCTGCCCATTTTTGCCTGATTTAAACAT
**QX_YfaU_I263K_fw 2**	GGCAAAAATGGGCCACGCAAAAAAGGTAGTTATTAAGCT
**QX_YfaU_I263K_rv 2**	AGCTTAATAACTACCTTTTTTGCGTGGCCCATTTTTGCC
